# Transcriptomic profiling of proteases and antiproteases in the liver of sexually mature hens in relation to vitellogenesis

**DOI:** 10.1186/1471-2164-13-457

**Published:** 2012-09-05

**Authors:** Marie Bourin, Joël Gautron, Magali Berges, Christelle Hennequet-Antier, Cédric Cabau, Yves Nys, Sophie Réhault-Godbert

**Affiliations:** 1 INRA, SIGENAE, UR83 Recherches Avicoles, 37380, F-37380 Nouzilly, France

**Keywords:** Proteases, Antiproteases, Liver, Sex steroids, Metabolism, Transcriptomics, Chicken egg, Vitellogenesis

## Abstract

**Background:**

Most egg yolk precursors are synthesized by the liver, secreted into the blood and transferred into oocytes, to provide nutrients and bioactive molecules for the avian embryo. Three hundred and sixteen distinct proteins have been identified in egg yolk. These include 37 proteases and antiproteases, which are likely to play a role in the formation of the yolk (vitellogenesis), as regulators of protein metabolism. We used a transcriptomic approach to define the protease and antiprotease genes specifically expressed in the hen liver in relation to vitellogenesis by comparing sexually mature and pre-laying chickens showing different steroid milieu.

**Results:**

Using a 20 K chicken oligoarray, a total of 582 genes were shown to be over-expressed in the liver of sexually mature hens (1.2 to 67 fold-differences). Eight of the top ten over-expressed genes are known components of the egg yolk or perivitelline membrane. This list of 582 genes contains 12 proteases and 3 antiproteases. We found that “uncharacterized protein LOC419301/similar to porin” (GeneID:419301), an antiprotease and “cathepsin E-A-like/similar to nothepsin” (GeneID:417848), a protease, were the only over-expressed candidates (21-fold and 35-fold difference, respectively) that are present in the egg yolk. Additionally, we showed the 4-fold over-expression of “ovochymase-2/similar to oviductin” (GeneID:769290), a vitelline membrane-specific protease.

**Conclusions:**

Our approach revealed that three proteases and antiproteases are likely to participate in the formation of the yolk. The role of the other 12 proteases and antiproteases which are over-expressed in our model remains unclear. At least 1/3 of proteases and antiproteases identified in egg yolk and vitelline membrane proteomes are expressed similarly in the liver regardless of the maturity of hens, and have been initially identified as regulators of haemostasis and inflammatory events. The lack of effect of sex steroids on these genes expressed in the liver but the products of which are found in the yolk suggests that these may be passively incorporated into the yolk rather than actively produced for that purpose. These results raise the question of the biological significance of egg yolk proteases and antiproteases, and more generally of all minor proteins that have been identified in egg yolk.

## Background

Egg yolk constitutes a major source of proteins, minerals, vitamins and lipids for the developing embryo. The major egg yolk proteins, with the exception of immunoglobulins, are synthesized by the liver of laying hens. Protein synthesis and lipogenesis are highly stimulated in the liver at sexual maturity in hens (15 to 20 fold) to support the incorporation of 0.75 kg proteins and 1.5 kg of triglycerides into the yolk over a year of commercial production. The production of estrogen and at a lower magnitude testosterone by the theca of the growing follicle increases 2 to 3 weeks before the production of the first egg and contributes to the development of reproductive organs and the synthesis of egg constituents [1-6]. Once secreted from the liver into the blood, egg yolk precursors are conveyed to the ovarian follicle, which consists of an oocyte surrounded by layers of supportive tissues, the theca externa and the theca interna, containing embedded capillaries which are separated from the granulosa layer by a thick basal lamina [7,8].

The transfer of the major egg yolk proteins such as Very Low Density Lipoproteins (VLDL) containing essentially apovitellenin and apolipoprotein-B, but also vitellogenins and some other plasma proteins from the blood such as serum albumin to the interstitial fluid of the thecae is possible due to the presence of broad discontinuities in the capillary endothelium. The fluid in the theca spaces is then “filtered” by the basal lamina, which selectively permits the passage of particles depending on their size and their molecular charge [9]. These particles then pass between the cells composing the granulosa layer. The size of this intercellular space is rearranged depending on the stage of follicular growth [10] and may also selectively control the uptake of specific plasma molecules. The relatively narrow intercellular space of the primordial follicle allows ferritin and possibly other molecules of similar size to cross, whereas in the late stages of rapid follicular growth, the shape of granulosa cells must accommodate the increasing flow of egg yolk precursors that accumulate at the surface of the plasma membrane of the follicle [7]. Egg yolk precursors finally diffuse through the zona pellucida to reach multifunctional oocyte-specific receptors, which achieve the specific uptake of plasmatic precursors into the forming yolk [11].

Egg yolk precursors are further processed within the oocyte by the action of proteases, the activities of which are regulated by protease inhibitors (antiproteases) or autolysis, to produce the final yolk constituents. It is assumed that cathepsin D, an aspartate protease, plays a major role in the generation of mature egg yolk constituents [12,13]. Recently Mann et al. and Farinazzo et al. [14,15] published a large list of egg yolk proteins including at least 37 proteases and proteases inhibitors. Most of these were relatively minor yolk components. Pathway analysis of egg yolk proteins has underlined the presence of numerous proteases and antiproteases associated with coagulation and inflammatory processes [16]. These molecules are normally observed in blood and the biological significance of these molecules in egg yolk has not been investigated. The wide range of physiological functions of proteases and antiproteases suggests that egg yolk proteases and antiproteases may be involved in many different biological processes including tissue remodeling, fertilization, angiogenesis, haemostasis, morphogenesis and protein maturation. These processes might be important in the oocyte during the process of follicular growth or in the egg during embryonic development. To gain insight into the characterization of the proteases and antiproteases that are specifically associated with vitellogenesis, we analyzed the expression of proteases and antiproteases in the liver of laying hens. Based on the postulate that most egg yolk proteins are secreted by the liver and that their synthesis is stimulated at sexual maturity of hens, and to eliminate proteins which are secreted by the liver and that are unrelated to egg yolk formation (factors in hemostasis, carrier proteins, immune effectors), we compared the expression profiles from the liver of sexually mature hens (laying hens) with that of the liver of sexually immature pullets. We focused on genes that are over-expressed in the liver of laying hens since these are likely to be secreted and transferred to the egg yolk, in contrast to the under-expressed genes (although the latter can regulate the expression of the others).

The present study provides an original overview of hepatic proteases and antiproteases that may be involved in the formation of the egg yolk and the perivitelline membrane, in fertilization or that could play a significant role during embryonic development as key components of the egg yolk. It also emphasizes the complementarities of both proteomic and transcriptomic strategies to better define the relevance of proteins which are associated with a specific milieu, and sheds lights on new approaches that can be implemented to answer questions related to their predicted biological significance.

## Results/discussion

### Hepatic transcriptomic profiling of laying hens versus pre-laying pullets

**Figure 1:**
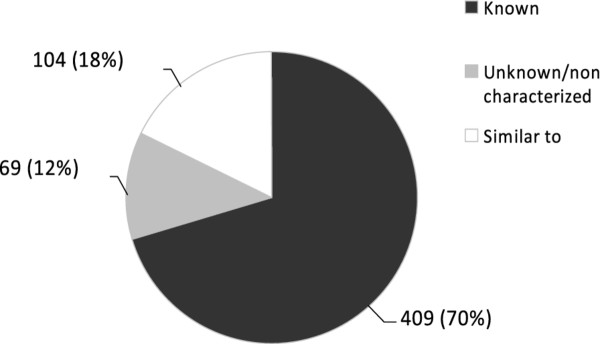
**Distribution of genes that are over-expressed in the liver of laying hens versus pre-laying pullets.** Numbers illustrate the numbers of genes that are known, unknown/uncharacterized or having a homolog in other species (similar to). Their relative percentages are indicated in parenthesis.

At sexual maturity of hens, hepatic gene expression is highly stimulated to support the metabolic changes associated with the development of the reproductive organs. Indeed, sexual maturity is partly characterized by the upregulation of gene expression and the expression of genes that only occurs in the presence of ovarian steroids to produce specific proteins in the liver, to sustain vitellogenesis. It is therefore not surprising that the statistical analysis of the results revealed that 582 genes (*P* < 0.01) are over-expressed in the livers of laying hens compared with livers of pre-laying pullets, with ratios ranging from 1.2 to 67. When analyzing the 582 over-expressed genes, we found that 70% of them were known, 18% have a homolog in other species and 12% are unknown (Figure 1). The functional annotation of all these over-expressed genes, using gene ontology annotation, text mining, pathway and network analyses will be the subject of a complementary article, in which we will integrate all these data to provide a unified view of the predicted biological pathways and functions associated with the hepatic metabolism of sexually mature hens (Réhault-Godbert et al., in preparation). The analysis of the top-ten over-expressed genes corroborates the relevance of our approach to study vitellogenesis: most of these ten genes are yolk components (GeneIDs 396449, 424534, 396476, 417848, 424547, 419301), one is found in the perivitelline membrane (GeneID:395418) which surrounds the egg yolk and one is clearly associated with lipogenesis (GeneID:421925) which occurs exclusively in the liver in birds (Table 1). The gene with the highest over-expression is ascribed to riboflavin-binding protein precursor (GeneID:396449, 67-fold over-expression), which stores and transports the vitamin ribloflavin into the yolk. Previous studies have reported that the expression of this gene is estrogen-dependent and that the riboflavin-binding protein is taken from the blood to enter the growing oocyte by receptor-mediated endocytosis, together with vitellogenins [17]. It has been shown that fertilized eggs from laying hens that are deficient in riboflavin-binding protein are unable to develop into viable embryos due to a severe impairment of fatty acid oxidation occurring after 10 days of incubation [18]. The list also includes two vitellogenins (GeneID:424534; 424547), which are the precursors of lipovitellins and phosvitin. These two vitellogenin-derived products result from intramolecular processing of vitellogenins by cathepsin D, after their transfer into the yolk [12]. Phosvitin is believed to store calcium, iron and other cations for the developing embryo [3]. We also identified apovitellenin-1 (GeneID:396476, Table 1), a low-density lipoprotein lipase inhibitor of egg yolk [19]. This apoprotein is specific to laying hens and prevents any alteration of the highly specific hen very low density lipoproteins (VLDL) from lipase activity during their transfer from the liver to the oocyte. Interestingly, we found two proteins with potential protease and protease inhibitor activity, respectively: “cathepsin E-A-like/similar to nothepsin” (GeneID:417848) and “uncharacterized protein LOC419301/similar to porin” (GeneID:419301, WAP four-disulfide core domain protein 3 precursor) that have been previously identified in the two proteomics surveys of the egg yolk [14,15]. The function of these proteins has not yet been explored.

**Table 1:** Top 10 over-expressed genes in livers of laying hens

**Protein name [*Gallus gallus*]**	**GeneID**	**Ratio ML/IML**	**Function (Uniprot)**
Riboflavin-binding protein precursor	396449	66.9	Required for the transport of riboflavin to the developing oocyte (future yolk)
Membrane-bound O-acyltransferase domain-containing protein 2	421925	50.7	Phospholipid biosynthetic process: Lysophospholipid acyltransferases catalyze the reacylation step of the phospholipid remodeling pathway also known as the Lands cycle
Vitellogenin-3 precursor	424534	45.9	Precursor of the egg-yolk proteins that are sources of nutrients during early development of oviparous organisms
Apovitellenin-1 precursor	396476	42.4	Protein component of the very low density lipoprotein (VLDL) of egg-laying females. Potent lipoprotein lipase inhibitor, preventing the loss of triglycerides from VLDL on their way from the liver to the growing oocytes
PREDICTED: cathepsin E-A-like/similar to nothepsin	417848	34.8	Unknown
Vitellogenin-1	424547	33.8	Precursor of the egg-yolk proteins that are sources of nutrients during early development of oviparous organisms
PREDICTED: dual 3',5'-cyclic-AMP and -GMP phosphodiesterase 11A	424133	24.1	Unknown (signal transduction, by homology)
PREDICTED: nebulin-related-anchoring protein	423899	21.1	Unknown (actin binding, by homology)
PREDICTED: uncharacterized protein LOC419301/similar to porin	419301	20.9	Unknown
Zona pellucida sperm-binding protein 1 precursor	395418	15.1	Major component of the peri-vitelline membrane

ML: livers of sexually mature hens; IML, livers of pre-laying pullets (immature pullets). Protein names were collected from NCBI sequence annotation that was available on 02-15-2012 (http://www.ncbi.nlm.nih.gov/protein).

Additionally, this list contains the membrane-bound O-acyltransferase domain-containing protein 2 (GeneID:421925, 51-fold over-expression), the activity of which is associated with phospholipid biosynthesis. This protein has been identified in chicken bursal lymphocytes [20]. By similarity, the membrane-bound O-acyltransferase domain-containing protein 2 is assumed to be integral to membranes and thus should not be secreted in the blood stream to be a constituent of the egg yolk. However, its predicted activity is consistent with the striking increase in lipogenesis (15 to 20 fold) that occurs in the liver with sexual maturation. In this report, we show for the first time that the expression of this protein is likely to be sex-steroid dependent.

Our data also revealed the presence of two predicted proteins, “PREDICTED: dual 3',5'-cyclic-AMP and -GMP phosphodiesterase 11A” (GeneID:424133, 24-fold increase) and “PREDICTED: nebulin-related-anchoring protein ”, which are highly expressed in the liver at sexual maturity of hens (GeneID:423899, 21-fold increase) and whose function remains to be explored. However, by similarity to mammals, “PREDICTED: dual 3',5'-cyclic-AMP and -GMP phosphodiesterase 11A” would have a role in signal transduction (Table 1). The last constituent of this list is Zona pellucida sperm-binding protein 1 precursor (GeneID:395418), which is over-expressed 15 fold in livers at sexual maturity of hens (Table 1). This hepatic protein is a known component of the perivitelline membrane, which is deposited in the ovary [21] after its synthesis and secretion by the liver of hens and is believed to play a central role in the initial interactions between spermatozoa and the oocyte [22].

### Hepatic proteases and antiproteases in laying hens

The analyses of the over-expressed genes related to proteases and antiproteases using Gene Ontology, Interpro and Uniprot databases revealed 18 genes (Table 2), which represent about 3% of the total over-expressed genes. This list includes 15 genes encoding proteases/peptidases and three genes encoding antiproteases (“uncharacterized protein LOC419301/similar to porin” (GeneID:419301), “papilin” (GeneID:428873) and “chymotrypsin inhibitor-like” (GeneID:768734). The expression of these 18 candidate genes was further verified by real-time q-RT-PCR. Log2 ratios of gene expression were used to perform the comparison between both approaches. As illustrated in Table 2, the over-expression was confirmed for 15 of the 18 candidates genes (*P* < 0.05). Three genes, PREDICTED: A disintegrin and metalloproteinase with thrombospondin motifs 5 (GeneID:427971, *P* = 0.11), PREDICTED: metalloendopeptidase OMA1, mitochondrial (GeneID:424670, *P* = 0.23) and “Signal peptide peptidase-like 2A precursor” (GeneID:415450, *P* = 0.46) were not significantly over-expressed in the liver of laying hens, when compared to the liver of pre-laying pullets by q-RT-PCR. Two of these three genes (“similar to OMA1 homolog”, a mitochondrial membrane protein, and “Signal peptide peptidase-like 2A”) encode proteins, which are predicted to be integral to the membrane [23,24] and should not be secreted into the blood to be constituents of the egg yolk. From these results, we decided to focus on the 15 genes that were shown to be over-expressed in the liver of laying hens in both the oligoarray study and q-RT-PCR analysis. Noticeably, all 15 genes correspond to predicted proteins. The gene ontology annotation of all these genes was therefore moderately informative (Table 3): most of these genes are associated with protein metabolism, as expected with molecules involved in proteolysis (protease) and its regulation (antiproteases). One of them is predicted to have function in embryonic development but also in peptide hormone and nerve growth factor processing (PREDICTED: Proprotein convertase subtilisin/kexin type 6, GeneID:395454). Members of the proprotein convertase family play a central role in the processing of various protein precursors ranging from hormones and growth factors to bacterial toxins and viral glycoproteins and the disruption of their genes have been associated with various metabolic disorders in mammals [25]. The mammalian homolog to inner mitochondrial membrane peptidase 2 isoform 2 (GeneID:417780) is known to catalyze the removal of transit peptides required for the targeting of proteins from the mitochondrial matrix, across the inner membrane, into the inter-membrane space. This protein has been described to have a role in fertility of both male and female mice [26]. However, this protein is reported to be integral to the mitochondrial membrane and might therefore have a role in the liver rather than at the ovarian site. Additionally, we identified two proteins that may be associated with cell growth and proliferation (glutamyl aminopeptidase, geneID:428771 and ubiquitin carboxyl-terminal hydrolase 3, geneID 415369) and one protease, aminopeptidase O (GeneID: 427467) with a potential role in the metabolism of fatty signaling molecules called leukotrienes, which are important agents in the inflammatory response. The role of aminopeptidase O in the metabolism of the liver at sexual maturity remains to be explored but it may maintain liver functionality in response to the dramatic increase in liver lipogenesis and protein biosynthesis during sexual maturity.

**Table 2:** List of over-expressed proteases and antiproteases in livers of laying hens

**Protein name [*Gallus gallus*]**	**GeneID/Ensembl transcript**	**Ratio ML / IML**		**Superfamily/domains**	**Activity**
		**Oligoarray**	**q-RT-PCR**		
PREDICTED: cathepsin E-A-like/similar to nothepsin	417848/ ENSGALT00000016271	34.8	31.1^b^	Peptidase A1	Aspartate protease
PREDICTED: uncharacterized protein LOC419301/similar to porin	419301/ ENSGALT00000011045	20.9	68.9^b^	Kunitz/WAP	Serine protease inhibitor
PREDICTED: ovochymase-2/similar to oviductin	769290/ ENSGALT00000008522	4.0	6.2^a^	Peptidase S1	Serine protease
PREDICTED: papilin	428873/ ENSGALT00000015156	2.7	3.9^b^	Kunitz	Serine protease inhibitor
PREDICTED: ubiquitin carboxyl-terminal hydrolase 3	415369/ ENSGALT00000005420	2.7	2.3^b^	Peptidase C19	Ubiquitinyl hydrolase
PREDICTED: aminopeptidase O	427467/ ENSGALT00000020596	2.1	1.8^a^	Peptidase M1	Aminopeptidase
PREDICTED: A disintegrin and metalloproteinase with thrombospondin motifs 17	415515/ ENSGALT00000011448	2.1	2.5^a^	Peptidase M12B	Zinc metallopeptidase
PREDICTED: proprotein convertase subtilisin/kexin type 6	395454/ ENSGALT00000011615	2.0	2.9^b^	Peptidase S8	Serine protease
PREDICTED: glutamyl aminopeptidase	428771/ ENSGALT00000014775	1.9	3.1^a^	Peptidase M28	Glutamate carboxypeptidase II-like
PREDICTED: N-acetylated alpha-linked acidic dipeptidase-like 2	429156/ENSGALT00000019779	1.9	1.7^b^	Peptidase M1	Aminopeptidase
PREDICTED: A disintegrin and metalloproteinase with thrombospondin motifs 5	427971/ ENSGALT00000025470	1.7	1.6^NS^	Peptidase M12B	Zinc metalloprotease
PREDICTED: chymotrypsin inhibitor-like	768734/ ENSGALT00000035386	1.7	1.6^a^	Kazal	Serine protease inhibitor
PREDICTED: OTU domain-containing protein 3	426746/ ENSGALT00000022924	1.6	1.9^b^	Peptidase C65	Cysteine protease
PREDICTED: TRAF3-interacting protein 1	424029/ ENSGALT00000006731	1.5	1.6^b^	Peptidase C19	Ubiquitinyl hydrolase
PREDICTED: metalloendopeptidase OMA1, mitochondrial	424670/ENSGALT00000017659	1.4	1.2^NS^	Peptidase M48	Zinc Metalloprotease
PREDICTED: mitochondrial inner membrane protease subunit 2 isoform 2	417780/ ENSGALT00000015446	1.4	2.1^b^	Peptidase S24/S26	LexA-related/ type I signal peptidase
PREDICTED: PPPDE peptidase domain- containing protein 2-like	770448/ ENSGALT00000019464	1.4	1.5^b^	DUF862/ PPPDE	Predicted thiol peptidase
Signal peptide peptidase-like 2A precursor	415450/ ENSGALT00000038799	1.3	0.9^NS^	Peptidase A22B	Signal peptide peptidase

For oligoarray analysis, statistical significance was determined as described in Methods. For Q-RT-PCR analysis, statistical significance was measured at 5% level (P < 0.05, ^a^) and whereas highly statistical significance was measured at 1% level (P < 0.01, ^b^). Non-significant results were denoted by ^NS^ (P > 0.05). Protein names were collected from NCBI sequence annotation that was available on 02-15-2012 (http://www.ncbi.nlm.nih.gov/protein). ML: livers of sexually mature hens; IML, livers of pre-laying pullets (immature pullets).

**Table 3:** Functional annotation of proteases and antiproteases which are over-expressed in the liver of laying hens

**Biological process**	**GeneID/ Protein accession number (RefSeq)**	**Protein name [*Gallus gallus*]**	**Related GO terms**	**Biological activity**
**Protein metabolism**	417780/XP_416025	PREDICTED: similar to inner mitochondrial membrane peptidase 2 isoform 2	GO:0006627	protein processing involved in protein targeting to mitochondrion; proteolysis
			GO:0006508	
	395454/XP_413892	PREDICTED: Proprotein convertase subtilisin/kexin type 6	GO:0009100	glycoprotein metabolic process; proteolysis; protein processing
			GO:0006508	
			GO:0016485	
	417848/XP_416090	PREDICTED: cathepsin E-A-like	GO:0006508	proteolysis
	429156/XP_426711	PREDICTED: N-acetylated alpha-linked acidic dipeptidase-like 2	GO:0006508	proteolysis
	415369/XP_413755	PREDICTED: ubiquitin carboxyl-terminal hydrolase 3	GO:0006511	ubiquitin-dependent protein catabolic process; regulation protein stability
			GO:0031647	
	427467/XP_001231898	PREDICTED: aminopeptidase O	GO:0006508	proteolysis
	799290/XP_001232535	PREDICTED: ovochymase-2	GO:0006508	proteolysis
	428771/XP_426327	PREDICTED: glutamyl aminopeptidase	GO:0006508	Proteolysis
**Multicellular organismal development, cell growth and proliferation**	395454/XP_413892	PREDICTED: Proprotein convertase subtilisin/kexin type 6	GO:0007354	zygotic determination of anterior/posterior axis, embryo; determination of left/right symmetry; secretion by cell
			GO:0007368	
			GO:0032940	
	428771/XP_426327	PREDICTED: glutamyl aminopeptidase	GO:0016477	cell migration; cell proliferation; cell-cell signaling
			GO:0008283	
			GO:0007267	
	415369/XP_413755	PREDICTED: ubiquitin carboxyl-terminal hydrolase 3	GO:0006281	DNA repair; histone deubiquitination; mitotic cell cycle
			GO:0016578	
			GO:0000278	
**Reproduction**	417780/XP_416025	PREDICTED: similar to inner mitochondrial membrane peptidase 2 isoform 2	GO:0001541	ovarian follicle development; spermatogenesis; ovulation
			GO:0007283	
			GO:0030728	
**Lipid and other metabolic process**	427467/XP_001231898	PREDICTED: aminopeptidase O	GO:0019370	leukotriene biosynthetic process
	395454/XP_413892	PREDICTED: Proprotein convertase subtilisin/kexin type 6	GO:0016486	peptide hormone processing; nerve growth factor processing; nerve growth factor production
			GO:0032455	
			GO:0032902	

To better appreciate which of these proteases and antiproteases are components of the egg, we compared this list of 15 candidates with proteases and antiproteases that were identified in the egg yolk by proteomics [14,15]. Thirty-seven protease and antiprotease candidates have been identified in these proteomic analyses (Tables 4 and 5). Most were present on the oligoarray but nine of them were missing (Tables 4 and 5). Q-RT-PCR on these missing candidates revealed that most of them were either not differentially expressed between the livers of sexually mature and immature hens (cystatin GeneID:396497, similar to complement component 2 GeneID:419574, Figure 2) or were over-expressed in the liver of immature pullets (similar to plasma protease C1 inhibitor GeneID:423132, coagulation factor X GeneID:415267, heparin cofactor II GeneID:395877 and similar to antithrombin GeneID:424440, Figure 2). Two genes were not found to be expressed in the liver regardless of the sexual maturity status (ovostatin GeneID:396151 and ovalbumin-related protein X GeneID:420898, data not shown) suggesting that these two antiproteases are expressed in the ovarian follicle (theca, granulosa cells) to be incorporated into the yolk during its growth. Regarding “Complement factor B-like protease”, we failed to find any gene or transcripts associated with this name and the corresponding IPI number (IPI00588708, [14]). Consequently, we do not have any information about the expression of this protease nor could we design the corresponding gene primers. When integrating both oligoarray and q-RT-PCR results, we show that only two of the genes which are over-expressed by the liver of laying hens are present in the egg yolk (Figure 3, Additional file 1: Tables S1). These two proteins correspond to “cathepsin E-A-like/similar to nothepsin” and “uncharacterized protein LOC419301/similar to porin”, the highest over-expressed proteins from the list of 15 candidates (Table 2). These proteins are also included in the top-ten over-expressed genes list of the global analysis (Table 1). “Cathepsin E-A-like/similar to nothepsin” shares a high protein sequence similarity with cathepsin D [14,27], another aspartic protease, that is involved in the maturation of yolk precursors [12]. Interestingly, some have shown that in Antarctic fish, nothepsin is uniquely expressed by the liver of females [28] and we revealed recently that “Cathepsin E-A-like/similar to nothepsin” has the same expression profile compared with fish nothepsin as it was found to be exclusively expressed in the liver of sexually mature hens [27]. The function of “cathepsin E-A-like/similar to nothepsin” in *Gallus gallus* has not yet been explored, but as a predicted aspartic protease it might assist cathepsin D in the processing of egg yolk precursors. “Uncharacterized protein LOC419301/similar to porin” is a predicted antiprotease containing one kunitz domain and nine whey-acidic protein domains. The presence of these multiple domains with potential inhibitory activities towards serine proteases suggests that this “uncharacterized protein LOC419301/similar to porin” could constitute a potent regulator of proteolytic activities within the egg yolk. This protein is related to “WAP four-disulfide core domain proteins”. Members of this family include elafin and secretory leucocyte protease inhibitor, which are involved in various aspects of mucosal immunity [29]. An alternative hypothesis is that “uncharacterized protein LOC419301/similar to porin” could be associated with egg yolk precursors to protect them from proteolytic activities/inactivation in the plasma, similarly to apovitellenin-1 that inhibits lipase activities preventing the loss of triglycerides from VLDL, on their way from the liver to the growing oocytes [19].

**Table 4:** **Proteases identified in the egg yolk **[14]**,**[15]** and the vitelline membrane (VM) **[30]

**Proteases name [*Gallus gallus*]**	**GeneID/Ensembl Transcript ID**	**Yolk proteome [**[14]**,**[15]**]**	**Vitelline membrane proteome [**[30]**]**	**Oligoarray ID**	**Ratio ML/IML (Transcriptome)**
56 kDa protein	428163/ENSGALT00000011138	+		RIGG00309	0.691
Aminopeptidase N (Ey)	395667/ENSGALT00000013470	+		RIGG14412	0.929
Anticoagulant protein C	395085/ENSGALT00000002745	+		RIGG07767	1.084
Coagulation factor IX	374258/ENSGALT00000010525	+		RIGG13402	1.020
*Coagulation factor X*	*415267/ENSGALT00000027175*	*+*		*Missing*	
*Complement factor B-like protease*	*-*^*(b)*^	*+*		*?*	
Hepatocyte growth factor like	396135/ENSGALT00000036285	+		RIGG07902	0.829
PREDICTED: cathepsin E-A-like/nothepsin	417848/ENSGALT00000016271	+		RIGG15447	34.846
Similar to carboxypeptidase D	417590/ENSGALT00000006838	+		RIGG12160	0.916
*Similar to complement component C2*	*419574/ENSGALT00000041167*	*+*		*Missing*	
Similar to Hepatocyte growth factor activator	422876/ENSGALT00000025185	+		RIGG09473	1.169
PREDICTED:Ovochymase-2/similar to oviductin	769290/ENSGALT00000008522		+	RIGG08201	4.014
Similar to plasminogen	421580/ENSGALT00000006829	+		RIGG08074	0.896
Similar to thrombin-activable fibrinolysis inhibitor	418851/ENSGALT00000027441	+		RIGG09638	1.018
Similar to transmembrane protease serine 13	419770/ENSGALT00000011876	+		RIGG05703	-^(a)^
Similar to transmembrane protease serine 9	428320/ENSGALT00000000620	+	+	RIGG07595	-^(a)^
Similar to ubiquitin specific protease 42	416431/ENSGALT00000005338	+		RIGG11623	-^(a)^
Thrombin	395306/ENSGALT00000013559	+		RIGG14442	0.978

ML: livers of sexually mature hens; IML, livers of pre-laying pullets (immature pullets).

^(a)^There was no statistical evidence of the expression of these genes in our transcriptomic approach. ^(b)^ No ensembl transcript or gene could be identified for this protein (ID IPI00588708) as reported in [2].

Proteases which were identified in the viltelline membrane and/or the egg yolk at a proteomic level but which were not spotted on the oligoarray are indicated in italics.

**Table 5:** **Antiproteases identified in the egg yolk **[14]**,**[15]** and the vitelline membrane (VM)**[30]

**Antiproteases name (*Gallus gallus)***	**GeneID/Ensembl Transcript ID**	**Yolk proteome [**[14]**,**[15]**]**	**Vitelline membrane proteome [**[30]**]**	**Oligoarray ID**	**Ratio ML/IML (Transcriptome)**
39 kDa protein	-^(b)^ /ENSGALT00000014597	+		RIGG05083	0.736
52 kDa protein	423433/ENSGALT00000017871	+		RIGG16020	0.745
*Cystatin*	*396497/ENSGALT00000014109*	*+*	*+*	*Missing*	
Follistatin	396119/ENSGALT00000024043		+	RIGG18223	0.979
*Heparin cofactor II*	*395877/ENSGALT00000002123*	*+*		*Missing*	
Metalloproteinase inhibitor 3	396483/ENSGALT00000020523		+	RIGG02367	0.389
Ovalbumin-related protein Y	420897/ENSGALT00000037191	+	+	RIGG09162	-^(a)^
*Ovalbumin-related protein X*	*420898/ENSGALT00000020997*	*+*	*+*	*Missing*	
Ovoinhibitor	416235/ENSGALT00000005545	+	+	RIGG11698	0.442
*Ovostatin*	*396151/ENSGALT00000037194*	*+*	*+*	*Missing*	
Similar to alpha-1 antitrypsin	423434/ENSGALT00000037577	+		RIGG01149	-^(a)^
Similar to alpha-2 plasmin inhibitor	417561/ENSGALT00000004728	+		RIGG11419	0.900
Similar to alpha2-macroglobulin	-^(b)^ /ENSGALT00000023051	+	+	RIGG17868	0.761
Similar to angiotensinogen	421543/ENSGALT00000018111	+		RIGG16106	1.092
*Similar to antithrombin*	*424440/ENSGALT00000007304*	*+*		*Missing*	
Similar to fetuin	424956/ENSGALT00000014007	+		RIGG14593	0.480
Similar to MGC68875	427942/ENSGALT00000000384	+		RIGG01854	0.220
Similar to mouse counterpart for human pigment epithelium derived factor	771608/ENSGALT00000004762	+		RIGG11435	0.802
Similar to ovomucoid	416236/ENSGALT00000005554	+	+	RIGG11701	-^(a)^
*Similar to ovulatory protein 2*	*-*^*(b)*^*/ENSGALT00000011044*		*+*	*Missing*	
*Similar to plasma protease C1 inhibitor*	*423132/ENSGALT00000011936*	*+*		*Missing*	
Similar to prekininogen	424957/ENSGALT00000014125	+		RIGG08639	1.160
Similar to putative porin	419301/ENSGALT00000011045	+		RIGG05232	20.869

ML: livers of sexually mature hens; IML, livers of pre-laying pullets (immature pullets).

(a) There was no statistical evidence of the expression of these genes in our transcriptomic approach. ^(b)^ No corresponding geneID could be found in databases.

Antiproteases which were identified in the viltelline membrane and/or the egg yolk at a proteomic level but which were not spotted on the oligoarray are indicated in italics.

**Figure 2:**
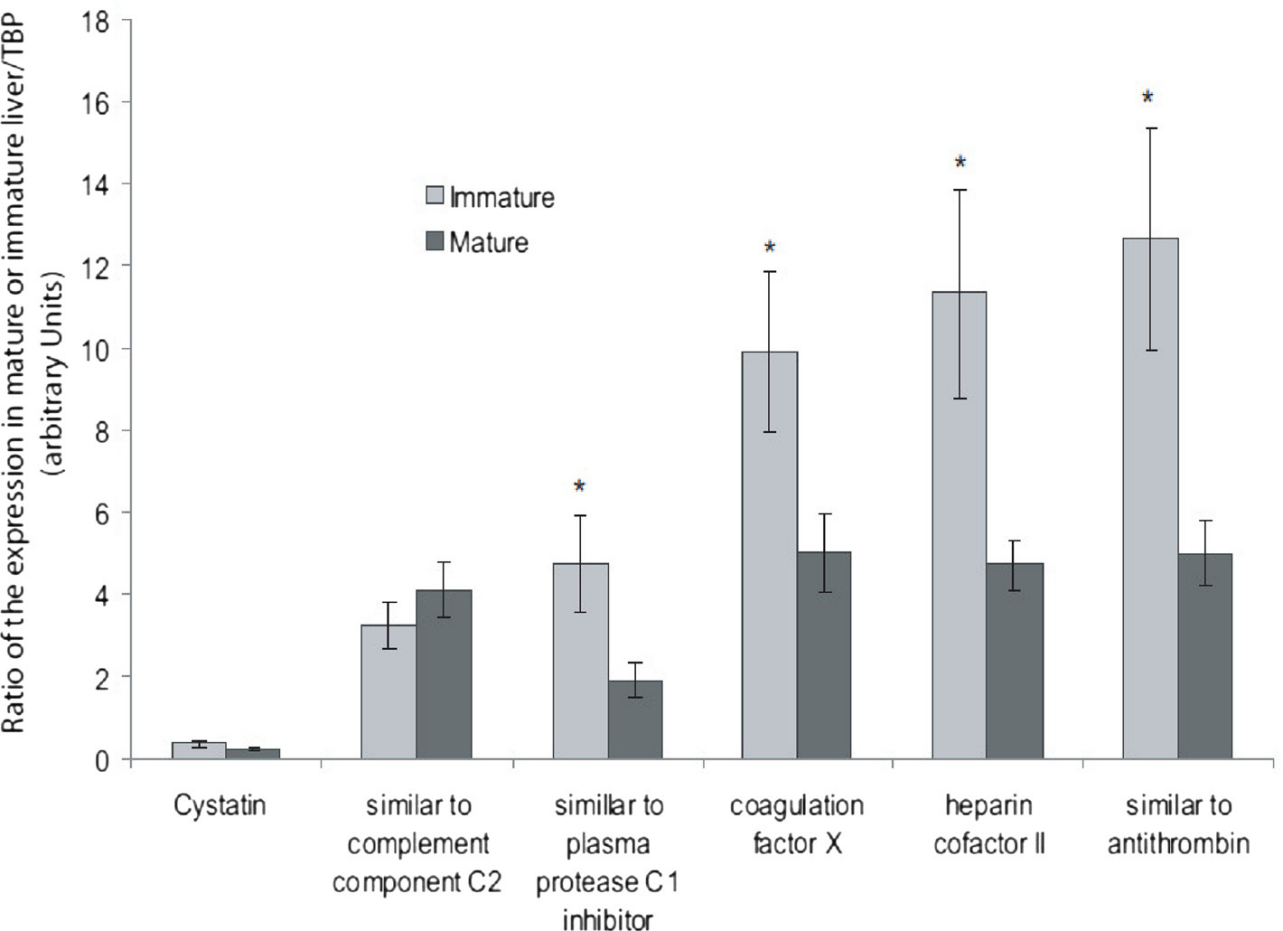
**Q-RT-PCR analysis of egg yolk proteases and antiproteases, which were not present on the oligoarray (Tables **4** and **5**).** Statistical significance was measured at 5% level (P < 0.05) and was denoted by an asterisk (*).

**Figure 3:**
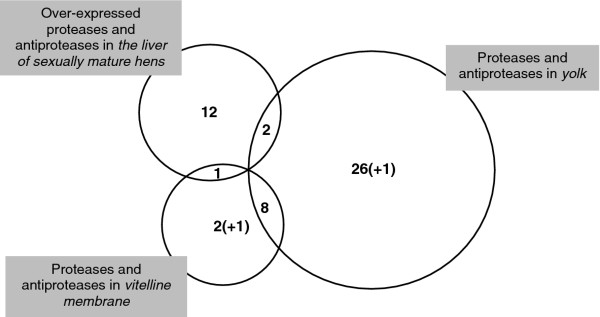
**Venn diagram of over-expressed proteases and antiproteases in the liver of laying hens compared to proteases and antiproteases identified in the yolk and the vitelline membrane (VM) (Tables **4** and **5**).** The numbers +1 indicated in parenthesis represent “similar to ovulatory protein 2” (vitelline membrane proteome) and “complement factor B-like protease” (egg yolk proteome), for which information is lacking.

Part of the perivitelline membrane is formed in the follicle and depends on the expression of proteins by theca and granulosa cells but proteins from the liver are also important [21]. In this context, we compared the list of our 15 candidates from the transcriptomic approach with that of proteases and antiproteases identified in the vitelline membrane’s proteome [30]. This revealed that “ovochymase-2/similar to oviductin”, which is over-expressed by the liver of laying hens (four-fold difference) is a protease that is specific to the vitelline membrane, as compared with the other egg compartments [30] (Table 4). This protease has been reported to be a key factor mediating sperm-binding to the oocyte, in Xenopus [31] and mammals [32]. It is responsible for the maturation/proteolysis of major glycoproteins composing the zona pellucida [32] but also proteins from the inner layer of the vitelline membrane of avian oocytes [33]. We show for the first time that the avian homolog of oviductin is expressed by the liver of laying hens and that its expression is likely to be sex-steroids dependant. Eight of the proteases and antiproteases identified in the vitelline membrane are also recovered in the egg yolk (Figure 3, Tables 4 and 5). Two of them are specific to the vitelline membrane, follistatin (GeneID:396119) and metalloproteinase inhibitor 3 (GeneID:396483) (Figure 3, Table 5) and are not differentially expressed by the liver in response to sex-steroid stimulation. No information of expression could be found for “Similar to ovulatory protein 2” as we failed to design functional primers to study its expression in the liver, in part due to the short length of the sequence available in databases (absence of geneID, Table 5).

To summarize, in the list of the 15 proteases and antiproteases over-expressed by the liver of laying hens by both oligoarray and q-RT-PCR, only three of them were actually known components of the egg yolk (“Cathepsin E-A-like/similar to nothepsin”, GeneID 417848 and “Uncharacterized protein LOC419301/similar to porin”, GeneID:419301) or the vitelline membrane (“ovochymase-2/similar to oviductin”, GeneID:769290). Further analyses were therefore needed to better appreciate the respective role of the other 12 proteases and antiproteases that are overexpressed in the liver of mature hens, in the process of egg yolk or vitelline membrane formation and also to explore the biological significance of all the other candidates present in the egg yolk including those not specifically expressed by the liver in relation to egg yolk formation.

To predict which of this list of 12 overexpressed proteases and antiproteases are secreted into the blood stream, we searched for the presence of signal peptides in their protein sequence (SignalP [34], Table 6). Only four of them were predicted to be secreted through the classical secretory pathway. However, it is noteworthy that some egg proteins do not possess a conventional signal peptide (the best example is probably ovalbumin, the major egg white protein) meaning that the restricted list of five potential secreted proteins as determined by SignalP might be slightly underestimated. Protein can also be secreted through unconventional pathways including direct translocation across plasma membranes and vesicle-mediated exocytosis [35]. Such unconventional pathways can be predicted using SecretomeP [36], a program that has been initially designed to predict protein secretion in mammals and bacteria. The use of this program to predict non classical secretory pathways in non-mammalian vertebrates, including *Gallus gallus*, has not been yet validated. However, when considering ovalbumin, a protein which does not possess a classical signal peptide and which is unambiguously secreted into the egg, it is predicted to be secreted through an unconventional secretory pathway using SecretomeP. These results suggest that SecretomeP can be informative to predict non-classical secreted proteins in *Gallus gallus*. Using this approach applied to the list of 15 genes, four have a signal peptide and six would be secreted through an unconventional pathway (Table 6). Four of these 15 proteins are predicted to be integral to the membrane by homology with their human counterpart (mitochondrial membrane peptidase 2 isoform 2) and using tools for predicting transmembrane domains (Table 6) [37]. Additionally, one of them is potentially localized in the nucleus (ubiquitin carboxyl-terminal hydrolase 3, Table 6). By combining both the literature on mammalian homologs and predictive tools for secretion and for transmembrane domains, we can propose a list of seven proteins that could potentially be secreted to be incorporated in the egg yolk or the vitelline membrane. These include “cathepsin E-A-like/similar to nothepsin”, “uncharacterized protein LOC419301/similar to porin” that were identified in the egg yolk and “ovochymase-2/similar to oviductin” which was found to be a component of the vitelline membrane. Additionally, our approach revealed at least 4 relevant new candidates (proprotein convertase subtilisin/kexin type 6, chymotrypsin-inhibitor-like, ubiquitin carboxyl-terminal hydrolase 30 and OTU domain-containing protein 3) that are over-expressed by the liver of sexually mature hens and could be potentially secreted into the blood stream to be transferred into the egg yolk or the vitelline membrane.

**Table 6:** Cellular localisation of proteases and antiproteases that are over-expressed in livers of laying hens (in both oligoarray and q-RT-PCR studies)

**Protein name [*Gallus gallus*]**	**GeneID/Ensembl transcript**	**Secretion [**[36]**]**	**Cellular localisation (transmembrane helice [**[37]**], nuclear localisation by homology)**
PREDICTED: cathepsin E-A-like/similar to nothepsin	417848/ENSGALT00000016271	Classical secretion (Signal peptide)	-
PREDICTED: uncharacterized protein LOC419301/similar to porin	419301/ENSGALT00000011045	Non-classical secretion	-
PREDICTED: ovochymase-2/similar to oviductin	769290/ENSGALT00000008522	Classical secretion (Signal peptide)	-
PREDICTED: papilin	428873/ENSGALT00000015156	-	-
PREDICTED: ubiquitin carboxyl-terminal hydrolase 3	415369/ENSGALT00000005420	-	Nuclear (by homology)
PREDICTED: aminopeptidase O	427467/ENSGALT00000020596	-	-
PREDICTED: A disintegrin and metalloproteinase with thrombospondin motifs 17	415515/ENSGALT00000011448	-	1 transmembrane domain
PREDICTED: proprotein convertase subtilisin/kexin type 6	395454/ENSGALT00000011615	Non-classical secretion	-
PREDICTED: glutamyl aminopeptidase	428771/ENSGALT00000014775	Non-classical secretion	1 transmembrane domain
PREDICTED: N-acetylated alpha-linked acidic dipeptidase-like 2	429156/ENSGALT00000019779	Non-classical secretion	1 transmembrane domain
PREDICTED: chymotrypsin inhibitor-like	768734/ENSGALT00000035386	Classical secretion (Signal peptide)	-
PREDICTED: OTU domain-containing protein 3	426746/ENSGALT00000022924	-	-
PREDICTED: TRAF3-interacting protein 1	424029/ENSGALT00000006731	Classical secretion (Signal peptide)	-
PREDICTED: mitochondrial inner membrane protease subunit 2 isoform 2	417780/ENSGALT00000015446	Non-classical secretion	Mitochondrial membrane (by homology)
PREDICTED: PPPDE peptidase domain-containing protein 2-like	770448/ENSGALT00000019464	Non-classical secretion	-

Additional studies will be needed to better appreciate the biological relevance of these four new candidate proteases and antiproteases as constituents of the egg yolk or the vitelline membrane. Some of these components might not be involved in vitellogenesis and instead contribute to liver homeostasis in response to the dramatic increase in lipogenesis and protein biosynthesis observed in liver of hens at sexual maturity. At least one protein of this limited list of four new candidates could have a role in hepatic protein catabolism as an ubiquityl hydrolase (similar to ubiquitin specific proteinase 40). In fact, it has been shown that ubiquitin specific peptidases are involved in the maintenance of genome integrity [38] and in the homeostasis of cellular proteins [39].

From the comparative analyses between the present transcriptomic approach and the published proteomic data, we emphasise the fact that the majority of egg yolk proteases and antiproteases are not differentially expressed in the liver of mature hens and immature pullets. Thirty-seven proteases and antiproteases were identified in egg yolk [14,15]. Most of these egg yolk proteases and antiproteases were initially defined as blood components playing a role in various plasmatic cascades including coagulation and fibrinolysis (Thrombin, Factor IX and X, Anticoagulant protein C, Similar to plasma protease C1 inhibitor, Similar to complement component C2, Heparin cofactor II, Similar to antithrombin, Hepatocyte growth factor like, Hepatocyte growth factor activator, plasminogen, Similar to alpha2-macroglobulin, Similar to alpha 2 plasmin inhibitor and Similar to angiotensinogen) [16,40-42]). This consideration suggests that all these components which are minor components of egg yolk [14,15], might not necessarily be actively incorporated into the yolk but rather be non-selectively engulfed by endocytic vesicles after interaction with specific egg yolk components (Very low density lipoproteins, Vitellogenins, Immunoglobulins Y, Alpha-2 macroglobulin, etc.) with their oocyte-receptor. In fact, it has been shown that plasma alpha-2 macroglobulin, an antiprotease displaying broad inhibitory activity, can interact with oocyte-receptors [43]. Therefore, most plasma proteases which are entrapped in a complex with alpha-2 macroglobulin could be internalized into the yolk together with the antiprotease. Consequently, alpha2-macroglobulin could serve as a carrier protein for plasma proteases [44]. It might be interesting to investigate the activity of these various proteases and antiproteases in egg yolk to assess whether they have a specific role in this compartment (angiogenesis for example), despite the low concentration found in proteomic approaches [14,15].

Additionally, eight of these thirty-seven proteases and antiproteases identified by proteomics were not found to be expressed by the liver regardless of sexual maturity: Similar to transmembrane protease serine 13, Similar to transmembrane protease serine 9, Similar to ubiquitin specific protease 42, Ovalbumin related protein Y, Similar to alpha-1 antitrypsin, Similar to ovomucoid (Tables 4 and 5, ^(a)^) and Ovalbumin-related protein X and Ovostatin (by q-RT-PCR, see text above). These results suggest that these egg yolk components might be expressed locally by other tissues than the liver, i.e., theca or granulosa cells.

Regarding the vitelline membrane, twelve proteases and antiproteases were identified in this compartment. As we mentioned before, one of them is over-expressed by the liver at sexual maturity (“ovochymase-2/similar to oviductin”). Regarding the five remaining candidates that are expressed by the liver, two of them are found to be not differentially expressed in our model (follistatin, Table 5 and cystatin, Figure 2) and in contrast, three are over-expressed in the liver of immature pullets : Metalloproteinase inhibitor 3, 2.5 fold-overexpression, Ovoinhibitor, 2.3 fold-overexpression, and Similar to alpha2-macroglobulin, 1.3 fold-overexpression, (Table 5). Five out of the twelve proteases and antiproteases that were identified in the vitelline membrane are not expressed by the liver (Ovalbumin-related protein Y, Ovalbumin-related protein X, Ovostatin, Similar to transmembrane protease serine 9, and Similar to ovomucoid, Tables 4 and 5). These results are consistent with the fact that the vitelline membrane is formed sequentially, first within the ovary and second in the infundibulum region of the oviduct [45]. Further studies study will be required to analyze the expression profile of proteases and antiproteases in the infundibulum and granulosa cells [46,47] to complete the source of the secreted proteases or antiproteases identified in the vitelline membrane [30]. Finally, additional analysis will also be needed to investigate the expression of “similar to ovulatory protein 2” in relation to the formation of vitelline membrane.

## Conclusions

In the last decade, the development of high throughput methods including proteomics with the development of chicken databases, thanks to the *Gallus gallus* genome sequencing project, have completely changed our view of the protein biochemistry of the egg. About 50 different proteins had been identified before the advent of these new approaches. To date, several hundred new egg constituents have been predicted in egg. When considering the egg yolk compartment alone, 316 distinct gene products have been identified, by proteomics. This list includes about ten major components, serum albumin, apovitellenin, ovalbumin, vitellogenins, immunoglobulins, apolipoprotein and avidin homologs, and many minor components, the function of which are not known [14,15]. Egg yolk proteins result mainly from the expression of their precursors by the liver. These precursors are secreted into the blood prior to their uptake by the oocyte in the yolk follicle. However, the liver expresses in the basal state many proteins such as hemostasic and fibrinolytic factors, carrier proteins and immune effectors which can be undiscriminately incorporated into the egg yolk by the endocytosis of egg yolk-specific proteins. In fact, the functional annotation of egg yolk proteins has revealed that many proteases and antiproteases identified in the egg yolk are known participants of coagulation/fibrinolysis cascades [16]. The biological significance of the presence of such molecules in the egg yolk is not known and with regards to their low concentration in this compartment, it is necessary to assess whether their presence is coincidental or necessary for the functional properties of the egg yolk. To address this question, we focused on the expression of egg yolk proteases and antiproteases in the liver of laying hens (high steroid milieu) and we compared this expression with that of the liver of pre-laying pullets (low steroid milieu) to remove proteins unrelated to egg yolk formation, using a transcriptomic approach. Results revealed 15 proteases and antiproteases, which are over-expressed by the liver at sexual maturity of hens. Only two of them are known constituents of the egg yolk (“cathepsin E-A-like/similar to nothepsin” and “uncharacterized protein LOC419301/similar to porin”) and one of them (“ovochymase-2/similar to oviductin”) was identified in the proteomic analysis of the vitelline membrane. Further studies will be needed to assess the mechanism by which “cathepsin E-A-like/similar to nothepsin” and “uncharacterized protein LOC419301/similar to porin”, and “ovochymase-2/similar to oviductin” are incorporated into the egg yolk and the vitelline membrane, respectively. The next challenge will consist of characterizing the biological activities of these three relevant proteins in the process of egg yolk formation/maturation and fertilization or after oviposition, to assist embryonic development. In addition, we have shown that most proteases and antiproteases which have been identified in egg yolk by proteomics are not specifically expressed by the liver in relation to the egg yolk formation. The expression of most proteases and antiproteases identified in egg yolk is independent of sex steroids, which suggests that these proteins may be passively incorporated in the yolk. Therefore, their biological significance and their related activity in the egg yolk will need to be demonstrated. Altogether, this work emphasises the complementarities of proteomics, transcriptomics and predictive tools to investigate the biological significance of proteins related to egg formation.

## Methods

### Animal handling and housing

Animals were bred at the experimental unit Pôle d’Expérimentation Avicole de Tours according to the European legislation on ‘the protection of animals used for experimental and other scientific purposes’ set by the European Community Council Directive of November 24, 1986 (86/609/EEC) and under the supervision of two authorized scientists S. Réhault-Godbert and J. Gautron (Authorizations # 37-144 and 7323, respectively). Animals were housed and fed according to the recommendations defined by the Institut National de la Recherche Agronomique (INRA). Eight ten-week old pre-laying pullets and eight thirty-eight-week old laying hens (ISA brown, Hendrix Genetics, St-Brieuc, France) were euthanized to collect the livers that were quickly frozen in liquid nitrogen and stored at -80°C until further use.

### RNA isolation

Total RNA was extracted from frozen tissue samples using a commercial kit RNA-L (Macherey-Nagel, Düren, Germany) according to the manufacturer's procedure, and simultaneously treated with DNase. RNA concentrations were measured at 260 nm and the integrity of RNA was evaluated on agarose 1% and with an Agilent 2100 Bioanalyzer (Agilent Technologies, Massy, France). RNA samples were kept frozen at -80°C until further use.

### Oligoarray hybridization

Only RNA samples with a 28S/18S ratio > 1.3 were considered for labeling and hybridization. Thirty micrograms of total RNA were used for labeling the cDNA with the Superscript® Plus Indirect cDNA Labelling System (Invitrogen, Cergy Pontoise, France). A balanced block design was used for hybridization where half of the sample were labeled with Alexa® 555 fluorescent dye and the other half with Alexa® 647 (Fisher Scientific BioBLock, Illkirch, France) using the Superscript® Plus Indirect cDNA Labelling System (Invitrogen, Cergy Pontoise, France). After synthesis and purification, the efficiency of the cDNA sample labeling as well as the quantity of labeled cDNA was determined using a Nanodrop ND 1000 (Nanodrop, Nyxor Biotech, Palaiseau, France) by measuring absorbance at 260, 320, 554, 650 and 750 nm. Samples were then hybridized on a micro-array ggallus_ARK-Genomics_20K (GEO: GPL6049) produced by Centre de Ressources Biologiques GADIE (INRA, Jouy-en-Josas, France). The features corresponding to the oligo-array are as follows. This 20.7 K chicken oligo array contains 20,676 features that include 20,460 oligos (about 70 mers) and 216 control spots of the Operon Production Tracking Oligo opHsV04NC000001, corresponding to 12595 different genes (Chicken oligo annotation, version 5 released on Oct 27th, 2009 by SIGENAE [48]). A dynamic hybridization was performed using 50 to 60 μl of PRONTO buffer (Corning, Life Sciences) containing 1 pmole/μl of each fluorescent probe and in a Slide-Booster hybridizer (Olympus Advalytix, Germany), at 42°C for 16 h as recommended by manufacturers. Oligoarrays were then washed using AdvaWash apparatus (Olympus Advalytix, Germany) and slides were then scanned using a GenePix 4000 B microarray scanner (Axon Molecular Devices, Sunnyvale, CA, USA). The GenePix Pro 6.0 software was used to acquire the fluorescent pictures, align the spots, quantify their intensity and finally export the GenePix Reports (GPR) files containing raw spot intensity. A total of eight microarrays slides corresponding to eight livers from immature pullets and eight livers from mature hens were hybridized. The GPR files were stored in the BioArray Software Environment (BASE) of SIGENAE [48] for further processing.

### Quantitative reverse transcriptase PCR

Five micrograms of total RNA samples that were used for microarrays experiments were reverse-transcribed using the superscript II kit (Invitrogen, Cergy Pontoise, France) and random hexamers (GE Healthcare, Uppsala, Sweden). Classical PCR was performed using primers for proteases and antiproteases (Additional file 1: Table S1) for 30 cycles at 60°C and resulting PCR products were submitted to sequencing for verification. Subsequently, cDNA sequences were amplified in real time using Sybr Green I Master kit (Roche, Mannheim, Germany) with the LightCycler 480 apparatus (Roche Diagnostics, Meylan, France). A melting curve program was carried out from 65 to 95°C in 1 min for each individual sample. Each run included triplicates of control cDNA corresponding to a pool of cDNA from all tissues. The control cDNA was diluted from 1:6.25 to 1:25,600 and relative arbitrary quantities were defined.

The threshold cycle (Ct), referred to as the Crossing point (Cp) on light cycler and defined as the cycle at which fluorescence rises above a defined base line, was determined for each sample and control cDNA. A calibration curve was calculated using the Cp values of the control cDNA samples and relative amount of unknown samples were deduced from this curve. To account for variations in mRNA extraction and reverse transcription reaction between samples, probe mRNA levels were corrected relative to Tata Binding Protein (TBP). Levels of Tata box binding mRNA were determined using Sybr Green reaction and the following primers 5’GCGTTTTGCTGCTGTTATTATGAG3’ (forward) and 5’TCCTTGCTGCCAGTCTGGAC3’ (backward).

### Statistical data analysis

#### Oligoarray

The profiling of differentially expressed genes in liver between mature and immature hens was determined using Anapuce R package [49]. Spot intensities were calculated using the median value that was logarithm transformed (log2). A normalization consisting of global locally-weighted regression (LOWESS) was applied on the overall intensity log ratio to remove the bias due to fluorescence incorporation. A block effect was also corrected by subtracting the median value of the block. Spot intensities were retained when present in at least 50% of samples. We performed a moderated t-test: rather than estimating within-group variability for each gene, we assume several groups of variance shared by similar genes using a mixture model VarMixt. We have chosen a unilateral statistical test to specifically evaluate genes that are over-expressed in the liver of mature hens compared to immature hens. Taking into account that all tests were simultaneous, p-values were adjusted by a Benjamini-Hochberg [50] multiple testing procedure to control the False Discovery Rate at level 1%. The microarray data was deposited in the NCBI Gene Expression Omnibus (GEO) data repository under series accession number GSE35595.

#### Q-RT-PCR

The log of the ratio sample/TBP RNA was used for statistical analysis using StatView version 5, software (SAS Institute Inc. Cary, NC). One-way analysis of variance was performed to detect differences (*P* < 0.05) in gene expression between livers of laying hens and livers of pre-laying pullets.

#### Bioinformatics analysis of overexpressed genes

Annotation of differentially-expressed transcripts was performed using the BioMart tool related to chicken contigs present in the SIGENAE database [51]. SIGENAE assemblies were carried out using chicken cDNA and EST sequences available in public databases. Functional annotations were completed using general chicken annotations in Ensembl [52] and Entrez Gene databases [53]. The identification of related transcripts to proteases and antiproteases was performed using Gene Ontology (GO) terms, Interpro [54] and Uniprot databases [55].

AgBase, a curated, open-source, Web-accessible resource for functional analysis of agricultural plant and animal gene products [56] was used to classify GO terms.

## Competing interests

The authors declare that they have no competing interests.

## Authors’ contributions

MCB was involved in designing and planning the study, collecting the tissues, in oligoarray hybridization, data analysis and in the writing of the manuscript. JG participated in the experimental design, collection of samples, data analysis and as an expert in transcriptomics. MB was involved in the RNA extraction and qRT-PCR experiments. CHA performed the statistical analyses of the data. CC from SIGENAE [48] was requested for his expertise in the use of bioinformatic tools to cross chicken databases and to perform the functional annotation of the genes. YN contributed to the design of the experiment, scientific analyses and discussions and actively participated in the writing of the paper. SRG was involved in the coordination of the study, in data analyses and integration and in the writing of the paper. All authors read the manuscript and contributed to the critical revision of the paper.

## Supplementary Material

Additional file 1 **Table 1.** List of primers used to analyse the expression of hepatic proteases and antiproteases by q-RT-PCR in laying hens *versus* pre-laying pullets. (PDF 18 kb) Click here for file
