# Supplementary material for: Transcriptomic profiling of proteases and antiproteases in the liver of sexually mature hens in relation to vitellogenesis
Source: BMC Genomics. 2012 Sep 5;13:457. doi: 10.1186/1471-2164-13-457 (PMC3495648; doi:10.1186/1471-2164-13-457)
Supplement: Additional file 1 — Table 1. List of primers used to analyse the expression of hepatic proteases and antiproteases by q-RT-PCR in laying hens versus pre-laying pullets. (PDF 18 kb) [file 1471-2164-13-457-S1.pdf]

Additional Table 1

Protein Name [*Gallus gallus*]	GeneID	Forward primers	Backward primers
PREDICTED: cathepsin E-A-like/similar to nothepsin	417848	CCACATCAGCTTCACCATTG	CGTCCCTGTGAGCAGATTTT
PREDICTED: uncharacterized protein LOC419301/similar to porin	419301	TGTGGAGGAGTGTGAGAGTGA	AGAGAGGCAGGCATGCAGAT
PREDICTED: ovochymase-2/similar to oviductin	769290	TCACTGCAGGAGAGCATGAC	TGCTGGCAAAACTGATGAAC
PREDICTED: papilin	428873	TGCAGAGTTTGATGGGACAG	GCACTTGTCCTCCTTCTTGG
PREDICTED: ubiquitin carboxyl-terminal hydrolase 3	415369	GTTGTGCATCATGGTTCAGG	TGTGTCCCGTGGACTTGATA
PREDICTED: aminopeptidase O	427467	CGGTTCCTGGCAAGTACATTA	GCCAGAAGTGTCAAGCCAAT
PREDICTED: A disintegrin and metalloproteinase with thrombospondin motifs 17	415515	CAGACAGAGGAAATGCGACA	CCCAGTGGAGAGCAGAAAAG
PREDICTED: proprotein convertase subtilisin/kexin type 6	395454	CTGCTCCTGTGATGGCTACA	GGTGACAATTTTCCGCTCAT
PREDICTED: glutamyl aminopeptidase	428771	GATTCTGGAAGCCATTTGGA	ATTGCCTGAATTCTGCATCC
PREDICTED: N-acetylated alpha-linked acidic dipeptidase-like 2	429156	ACTGGAATGGGACATGGAAA	GGTGGCTCTTTGGTGACTGT
PREDICTED: A disintegrin and metalloproteinase with thrombospondin motifs 5	427971	GGGACCATATGCTCTCCTGA	CAAGGCTTGGAAGCATCAAT
PREDICTED: chymotrypsin inhibitor-like	768734	AAGCTGGAAGAGGGGAACAG	TGACACTCCCCATCACTCAA
PREDICTED: OTU domain-containing protein 3	426746	CCAGGAGACGGTGGAATACA	TGTCAGTGCCTTGAATCTGC
PREDICTED: TRAF3-interacting protein 1	424029	TCTCTCCAGTGCCTGAGGAT	TGCCAGCTGTCACCTGATAA
PREDICTED: metalloendopeptidase OMA1, mitochondrial	424670	ACTGTTGGTGTTCGGAAAGG	ATGGATAGCCCAGGTGAGTG
PREDICTED: mitochondrial inner membrane protease subunit 2 isoform 2	417780	GCATATTTGGGTGGAAGGTG	TGCTCTCTCTGGAGAGGTTTG
PREDICTED: PPPDE peptidase domain-containing protein 2-like	770448	TCTTTCTTCCTTGGGGGAGT	GCCGTAAAGCTTGTCCAAAA
Signal peptide peptidase-like 2A precursor	415450	GATGCAGCTCCTGGAATAGC	ACTTCTCATCCCCCACCTCT
Coagulation factor X	415267	CTTGCTCCTGTTTGGATGGT	GCCAGCTCATACCCACTTGT
Similar to complement component C2	419574	GCCTCTGTGAAGGATGTGGT	CTGTGGCATGGATTGAAGGT
Cystatin	396497	ACAACTTGCCCCAAGTCATC	GGCAGCGATACAATCCATCT
Heparin cofactor II	395877	GCAGAAGAGCATGACGAACA	AAAGAGGCATGAATCCAACG
Ovalbumin-related protein X	420898	TCCGTGAACATCCACCTACTCT	GGCTTGGTCTGATGCTGTTT
Ovostatin	396151	ACTGGGAGCAGAACCAGAGA	ATAAGCAGCAAGGGCTTGAA
Similar to antithrombin	424440	CTTCTTCGCCAAGCTCAACT	CTTCTGTAATGCGCCTCTCC
Similar to plasma protease C1 inhibitor	423132	GTCTGGTCGCTGCAGACTTT	GTCCCATGAAGAGAGGGATG
